# Antimicrobial Susceptibility Testing for Colistin: Extended Application of Novel Quantitative and Morphologic Assay Using Scanning Electron Microscopy

**DOI:** 10.1155/2024/8917136

**Published:** 2024-05-25

**Authors:** Omar Zmerli, Sara Bellali, Gabriel Haddad, Rim Iwaza, Akiko Hisada, Erino Matsumoto, Yusuke Ominami, Didier Raoult, Jacques Bou Khalil

**Affiliations:** ^1^Institut Hospitalo-Universitaire Méditerranée-Infection, Marseille, France; ^2^Aix-Marseille Université, Institut de Recherche pour le Développement (IRD), UMR Microbes Evolution Phylogeny and Infections (MEPHI), Marseille, France; ^3^Hitachi, Ltd., Research & Development Group, Tokyo, Japan; ^4^Hitachi High-Tech Corporation, 882 Ichige, Hitachinaka-shi, Ibaraki-ken 312-8504, Japan; ^5^Consulting Infection Marseille, 16 Rue de Lorraine, Marseille, France

## Abstract

**Background:**

Colistin (Polymyxin E) has reemerged in the treatment of MDR Gram-negative infections. Traditional Colistin AST methods have long turnaround times and are cumbersome for routine use. We present a SEM-AST technique enabling rapid detection of Colistin resistance through direct observation of morphological and quantitative changes in bacteria exposed to Colistin.

**Methods:**

Forty-four Gram-negative reference organisms were chosen based on their Colistin susceptibility profiles. Bacterial suspensions of ∼10^7^ CFU/mL were exposed to Colistin at EUCAST-ECOFF, with controls not exposed, incubated at 37°C, and then sampled at 0, 15, 30, 60, and 120 minutes. Phosphotungstic Acid (PTA) staining was applied, followed by SEM imaging using Hitachi TM4000PlusII-Tabletop-SEM at ×2000, ×5000 and ×7000 magnifications. Bacterial viability analysis was performed for all conditions by quantifying viable and dead organisms based on PTA-staining and morphologic changes.

**Results:**

We identified a significant drop in the percentage of viable organisms starting 30 minutes after exposure in susceptible strains, as compared to nonsignificant changes in resistant strains across all tested organisms. The killing effect of Colistin was best observed after 120 minutes of incubation with the antibiotic, with significant changes in morphologic features, including bacterial inflation, fusion, and lysis, observed as early as 30 minutes. Our observation matched the results of the gold standard-based broth microdilution method.

**Conclusions:**

We provide an extended application of the proof of concept for the utilization of the SEM-AST assay for Colistin for a number of clinically relevant bacterial species, providing a rapid and reliable susceptibility profile for a critical antibiotic.

## 1. Introduction

Antimicrobial resistance has been challenging the world for decades, with continuous attempts to control the spread of resistance through antimicrobial stewardship and enhanced clinical diagnostics. The arsenal of antimicrobials has also shifted, with the introduction of novel agents and the revival of old molecules that were previously abandoned due to toxicity and dosing difficulties [1]. One of these molecules, Colistin (Polymyxin E), has reemerged as a “last resort” agent to be used in the treatment of infections caused by multidrug resistant (MDR) Gram-negative pathogens. In particular, the recent increase in Carbapenem-resistant organisms (CRO) led to an unprecedented increase in the use of Colistin, as most available antibiotics, including novel molecules, do not provide adequate coverage in all cases [2]. Otherwise, many Gram-negative bacilli (GNB) remain susceptible to Colistin, except few genera such as *Proteus*, *Serratia*, and *Morganella*, which possess intrinsic resistance through a variety of complex mechanisms [3]. Colistin is a bactericidal antibiotic that exerts its effect primarily through disruption of the bacterial cell membrane through its interaction with the lipopolysaccharide layer (LPS), which eventually leads to bacterial death [4, 5]. Given its effectiveness, Colistin has also found its way into the veterinary and agricultural fields for both therapeutic and prophylactic uses [4]. Subsequently, Colistin resistance has progressed, induced by numerous mechanisms: chromosomally encoded resistance, mobile resistance, mutations, adaptation, and others that remain enigmatic to this day [3]. This presents complex challenges involving the use of Colistin in therapeutic settings, requiring rapid and accurate detection of pathogen susceptibility to the drug. Traditional antimicrobial susceptibility testing (AST) methods, in addition to having turnaround times extending to 72 hours [5], have often failed for Colistin, mainly due to its chemical properties [6]. The gold standard recommended by both CLSI and EUCAST is broth microdilution (BMD) [7], which involves a lengthy and operator-dependent protocol that is considered cumbersome for the daily workload of most laboratories. Other semiautomated and automated assays have been tested, with variable and suboptimal results [5, 8, 9].

We have recently reinstated the use of scanning electron microscopy (SEM) in microbiological diagnostic applications and recently described the success of using SEM in AST applications for imipenem [10, 11], vancomycin [12], and Colistin [11], demonstrating the feasibility of performing AST through morphological analysis. We have also demonstrated the utility of Phosphotungstic Acid (PTA) staining using SEM for quantitative analysis of the bacterial viability status after imipenem and Colistin treatments [11]. Therefore, we aim to apply the proof of concept of our SEM-AST technique on a wider scale to demonstrate an accessible, simplified, and rapid detection of Colistin resistance, considering morphologic and quantitative changes in bacteria exposed to Colistin and directly observed using SEM on glass slides, thus bypassing the limitations of traditional assays affected by the unique chemical structure of the Colistin molecule.

## 2. Materials and Methods

### 2.1. Antibiotic and Media Preparation

We used Colistin Sulfate (Sigma-Aldrich) dissolved in sterile distilled water (Bio-Rad Laboratories, Inc.) to prepare the antibiotic solution to be used according to the EUCAST concentrations (ECOFF) [13] for each bacterial strain (Supplementary Table S1). The antibiotic solution was freshly prepared for each experiment. Mueller–Hinton Broth II (MHB) (Millipore, Sigma-Aldrich) was prepared according to the manufacturer's instructions [14] and filtered at 0.22 *µ*m.

### 2.2. Bacterial Strain Selection

We selected 44 clinically relevant Gram-negative organisms from the available reference species in the “*Collection de Souches de l'Unité des Rickettsies*” (*CSUR*) collection. We verified the identity of the selected strains using MALDI-TOF MS (matrix-assisted laser desorption/ionization time-of-flight mass spectrometry; Bruker Daltonics, Germany) [15] and then performed antimicrobial susceptibility testing for all isolates using the UMIC® Colistin kit (Biocentric, Bandol, France) [16], selected as a suitable surrogate for the gold standard broth microdilution method (BMD) described in EN ISO 20776-2. A complete listing of selected reference strains, MIC values, and Colistin susceptibility is detailed in Table 1.

### 2.3. Sample Preparation

We prepared fresh solid cultures of the chosen isolates by first incubating them on Columbia agar + 5% sheep blood (bioMérieux, France) overnight and then resuspending them in 5 ml of 0.2 *µ*m filtered MHB at 37°C. We adjusted all bacterial suspensions to ∼10^7^ CFU/mL, corresponding to an optical density (O.D._600_) of 0.2 measured using the Ultrospec 10 cell density meter (Biochrom, UK) at a wavelength of 600 nm.

**Figure 1:**
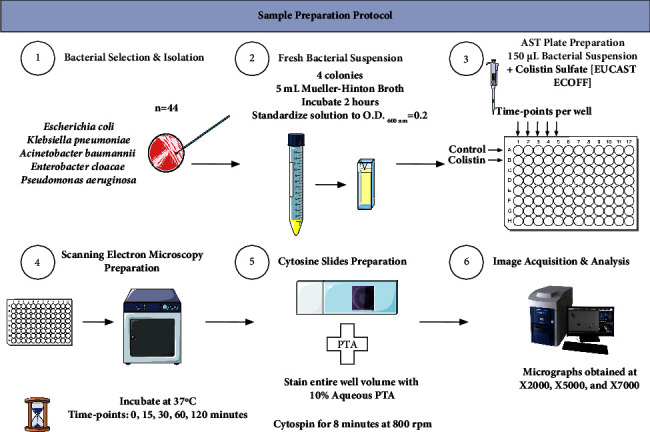
Workflow of the SEM-AST assay detailing the steps for sample preparation from culture to SEM observation.

We then divided the bacterial suspension into 96-well microplates (150 *µ*L/well), adding Colistin to the first row and leaving the second row without Colistin as our control. Colistin concentration was based on EUCAST guidelines [13]. We then incubated the plates at 37°C with shaking at 130 rpm, with progressive sampling of the wells at the following times: 0, 15, 30, 60, and 120 minutes. At each time point, we applied the Phosphotungstic Acid (PTA) staining methodology we developed for the discrimination of live bacteria from dead bacteria based on their contrast in SEM imaging [11, 17]. In brief, PTA is used as a viability stain for bacteria, based on its localization around the bacterial cell (for live organisms-dark contrast) and inside the bacterial cell (for dead organisms-bright contrast). Therefore, at each time point, we stained each well for 5 minutes with 50 *µ*L of 10% aqueous 10% Phosphotungstic Acid (PTA) (Sigma-Aldrich, St. Louis, MO, USA) at pH 7. We then used all the contents of the well to prepare the glass slides using Cytospin® (Thermo Scientific Shandon) at 800 rpm for eight minutes. We then used the TM4000PlusII tabletop scanning electron microscope (SEM) (Hitachi High-Tech, Japan) to obtain micrographs, with the following settings: 10 kV–15 kV accelerating voltage and BSE detector. We acquired the micrographs at ×2000, ×5000 and ×7000 magnifications, while using identical settings per condition. The acquisition settings are visible on each micrograph in the following format: instrument, accelerating voltage, working distance, magnification, and detector. We performed our experiment in triplicate for each of the chosen species. The entire process from sample to image and result read out is summarized in Figure 1.

### 2.4. Bacterial Viability Analysis and Quantification

To identify the viability status of observed PTA-stained bacterial cells on acquired micrographs, we performed a direct manual quantification of live and dead organisms on all acquired SEM micrographs, by means of a manual counting method categorizing 500 consecutively counted bacteria, per well/condition, into live and dead according to visible contrast difference (dark/bright).

In addition, PTA staining allowed us to visually track the morphological changes after antibiotic exposure and most importantly define ultrastructural changes in bacterial cells, not limited to cell inflation, fusion, deformation, and lysis. The count was performed using the Multipoint tool of ImageJ software [18].

### 2.5. Independent Operator Comparison

A comparison was made on randomly selected isolates with different susceptibility profiles between the gold standard broth microdilution method and our SEM-AST assay. We compared the readout of susceptibility results on experiments performed by two blinded independent operators.

### 2.6. Statistical Analysis

Manual quantification data consisting of the total count of 500 bacteria per isolate/experimental condition were percentage transformed and reported as means ± standard deviation for each isolate. The mean was then analyzed using one-way ANOVA followed by the post hoc Tukey test performed using GraphPad Prism 9.0 software (GraphPad, San Diego, CA). The differences between live and dead bacteria were statistically significant when the *p* value <0.05.

## 3. Results

### 3.1. Colistin-Resistant Strains

**Figure 2:**
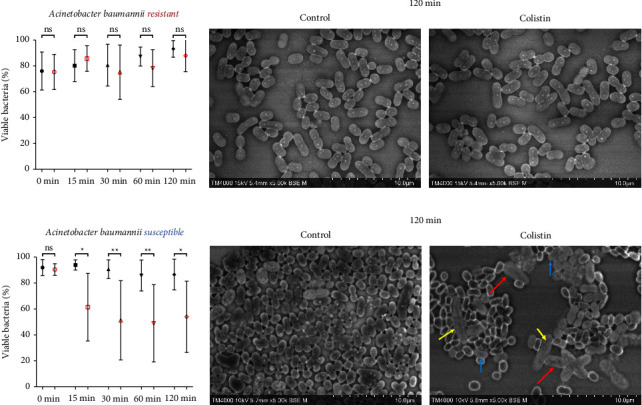
Colistin SEM-AST assay applied to Colistin-sensitive and Colistin-resistant strains of Acinetobacter baumannii. (a) Evolution of the percentage of viable bacteria for Colistin-resistant strains exposed to Colistin for 120 minutes. (b) SEM images of Colistin-resistant strains exposed to Colistin for 120 minutes. (c) Evolution of the percentage of viable bacteria for Colistin-sensitive strains exposed to Colistin for 120 minutes. (ns: not significant / ^∗^: *p* ≤ 0.05 / ^∗∗^: *p* ≤ 0.01) (d) SEM images of Colistin-sensitive strains exposed to Colistin for 120 minutes demonstrating morphologic changes (dysmorphism (red arrow), inflation (blue arrow), and fusion (yellow arrow)).

**Figure 3:**
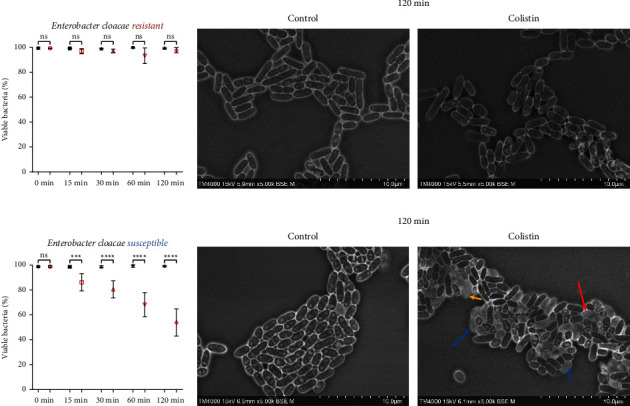
Colistin SEM-AST assay applied to Colistin-sensitive and Colistin-resistant strains of Enterobacter cloacae. (a) Evolution of the percentage of viable bacteria for Colistin-resistant strains exposed to Colistin for 120 minutes. (b) SEM images of Colistin-resistant strains exposed to Colistin for 120 minutes. (c) Evolution of the percentage of viable bacteria for Colistin-sensitive strains exposed to Colistin for 120 minutes. (ns: not significant / ^∗∗∗^: *p* ≤ 0.001/ ^∗∗∗∗^: *p* ≤ 0.0001) (d) SEM images of Colistin-sensitive strains exposed to Colistin for 120 minutes demonstrating morphologic changes (dysmorphism (red arrow), inflation (blue arrow), and lysis (orange arrow)).

**Figure 4:**
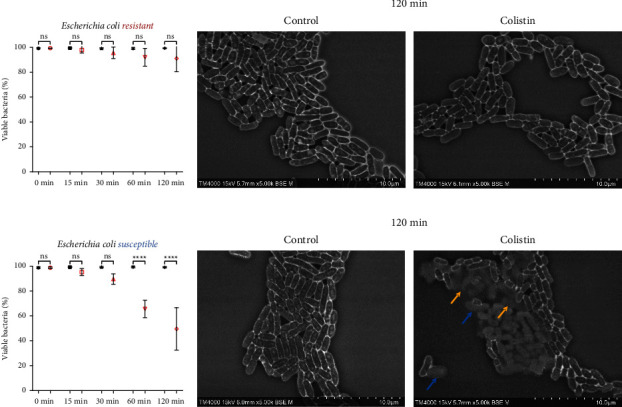
Colistin SEM-AST assay applied to Colistin-sensitive and Colistin-resistant strains of E. coli. (a) Evolution of the percentage of viable bacteria for Colistin-resistant strains exposed to Colistin for 120 minutes. (b) SEM images of Colistin-resistant strains exposed to Colistin for 120 minutes. (c) Evolution of the percentage of viable bacteria for Colistin-sensitive strains exposed to Colistin for 120 minutes. (ns: not significant / ^∗∗∗∗^: *p* ≤ 0.0001) (d) SEM images of Colistin-sensitive strains exposed to Colistin for 120 minutes demonstrating morphologic changes (inflation (blue arrow) and lysis (orange arrow)).

**Figure 5:**
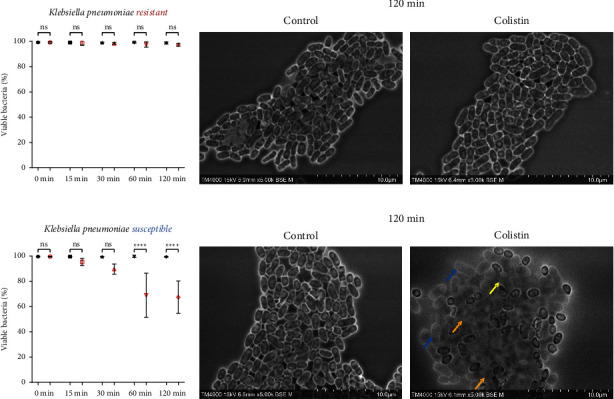
Colistin SEM-AST assay applied to Colistin-sensitive and Colistin-resistant strains of *K. pneumoniae*. (a) Evolution of the percentage of viable bacteria for Colistin-resistant strains exposed to Colistin for 120 minutes. (b) SEM images of Colistin-resistant strains exposed to Colistin for 120 minutes. (c) Evolution of the percentage of viable bacteria for Colistin-sensitive strains exposed to Colistin for 120 minutes. (ns: not significant / ^∗∗∗∗^: *p* ≤ 0.0001) (d) SEM images of Colistin-sensitive strains exposed to Colistin for 120 minutes demonstrating morphologic changes (inflation (blue arrow), fusion (yellow arrow), and lysis (orange arrow)).

**Figure 6:**
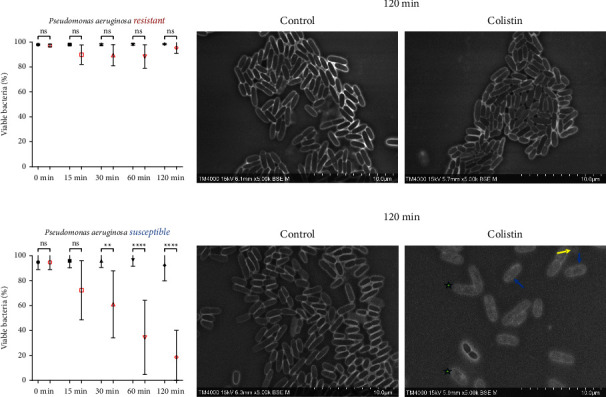
Colistin SEM-AST assay applied to Colistin-sensitive and Colistin-resistant strains of *P. aeruginosa*. (a) Evolution of the percentage of viable bacteria for Colistin-resistant strains exposed to Colistin for 120 minutes. (b) SEM images of Colistin-resistant strains exposed to Colistin for 120 minutes. (c) Evolution of the percentage of viable bacteria for Colistin-sensitive strains exposed to Colistin for 120 minutes. (ns: not significant / ^∗∗^: *p* ≤ 0.01 / ^∗∗∗∗^: *p* ≤ 0.0001) (d) SEM images of Colistin-sensitive strains exposed to Colistin for 120 minutes demonstrating morphologic changes (inflation (blue arrow), lysis (yellow arrow), and a granular pattern (green star)).

Colistin-resistant strains did not show any significant morphologic or quantitative change in Colistin-treated wells compared to controls. They demonstrated persistent morphology with no contrast change despite exposure to Colistin at all time points. The morphology appears almost identical at the 120-minute time point for *A. baumannii* (*n* = 3) (Figures 2(a) and 2(b)), *E. cloacae* (*n* = 5) (Figures 3(a) and 3(b)), *K. pneumoniae* (*n* = 5) (Figures 4(a) and 4(b)), *E. coli* (*n* = 4) (Figures 5(a) and 5(b)), and *P. aeruginosa* (*n* = 4) (Figures 6(a) and 6(b)).

### 3.2. Colistin-Sensitive Strains

Without exception, Colistin-susceptible strains for all tested species demonstrated a variation in the mean percentage of viable bacteria in Colistin-treated wells as compared to controls, with significant changes observed at different time points, accompanied with an array of pronounced morphologic changes.

#### 3.2.1. *Acinetobacter baumannii*

The mean percentage of viable bacteria in Colistin-treated wells at 15 minutes was significantly lower (61.45%) for all tested strains (*n* = 3) compared to controls (93.88%) (Figure 2(c)). This decrease was more distinct at 120 minutes, from 86.54% in the control to 53.96% in the Colistin-treated wells. On the morphological level, the difference in contrast was observed starting 15 minutes, with a clear change in shape and contrast of dead cells (white contrast) compared to live cells (dark contrast) (Figure 2(d)). Live cells appeared to have a more conserved morphology throughout the successive time points, while changes in dead cells were well defined starting the 30-minute time point, with dysmorphic (red arrow), inflated (blue arrow), and fused cells forming elongated structures (yellow arrow).

#### 3.2.2. *Enterobacter cloacae*

A significant decrease in the mean percentage of viable bacteria in Colistin-treated wells compared to controls was detected as early as 15 minutes in all tested strains (*n* = 5). The mean percentage dropped from 98.55% in the control to 85.15% in the Colistin-treated wells at 15 minutes (*p* < 0.05), and from 99.08% to 53.74% at 120 minutes, respectively (*p* < 0.05) (Figure 3(c)). Morphologically, contrast changes reflecting viability were clear at 15 minutes, but were most pronounced at 60 minutes. Similarly, dysmorphic bacterial cells were more visible at 60 minutes, with inflated (blue arrow) and disfigured (red arrow) morphologies. Interestingly, completely disintegrated bacterial cells with phantom-like appearance were visible, having the same contrast as dead bacteria (orange arrow) (Figure 3(d)).

#### 3.2.3. *Escherichia coli*

Early changes at 15 and 30 minutes were not statistically significant among the tested strains (*n* = 4). However, the mean percentage of viable bacteria decreased significantly 60 minutes after Colistin exposure, from 98.6% in the control to 65.48% in the Colistin-treated wells (*p* < 0.05). A more marked drop was clear at 120 minutes, from 98.69% to 52.11%, respectively (Figures 4(c) and 4(d)). A contrast difference consistent with bacterial death was apparent at the 30-minute time point for all tested strains. Thereafter, distorted morphologies were witnessed among dead bacterial cells, including inflated cells (blue arrow). Severe morphologic changes were noticeable at the 120 minute time point, with near obliteration of the original morphology of the bacterial cells and complete lysis (Figure 4(d); orange arrows).

#### 3.2.4. *Klebsiella pneumoniae*

All strains (*n* = 5) demonstrated parallel dynamics with a slight decrease in the mean percentage of viable bacteria starting at 30 minutes, from 99.59% in the control to 89.70% in the Colistin-treated wells, followed by a significant decrease at 60 minutes, from 99.59% to 89.7%, respectively (*p* < 0.05). This drop continued to reach 67.47% at 120 minutes after exposure to Colistin (Figure 5(c)). Contrast changes indicating bacterial cell death were seen at 30 minutes, with apparent morphologic distortion including inflation (blue arrow), fusion (yellow arrow), and complete lysis/phantom-like appearance at the latest time point (orange arrow) (Figure 5(d)).

#### 3.2.5. *Pseudomonas aeruginosa*

At 30 minutes, a significant decrease in the mean percentage of viable bacteria for all tested strains (*n* = 5) was observed, as compared to the controls. The decrease was most significant at 120 minutes, dropping from 95.74% in the control to 18.56% in the Colistin-treated wells (*p* < 0.05) (Figure 6(c)). Severe structural modifications were observed very early at the 30 minute time point, including the contrast change associated with dead bacterial cells and inflated (blue arrow) bacterial cells. Complete bacterial lysis (yellow arrow) was observed in micrographs obtained at 120 minutes after exposure to Colistin, in addition to the appearance of nonidentifiable debris and a very low percentage of viable bacteria (Figure 6(d)). Furthermore, a particular granularity was visible in dead bacterial cells killed by Colistin, compared to dead cells normally encountered in the control group (green star) in all of the tested strains.

#### 3.2.6. Global View on Colistin-Susceptible Isolates

**Figure 7:**
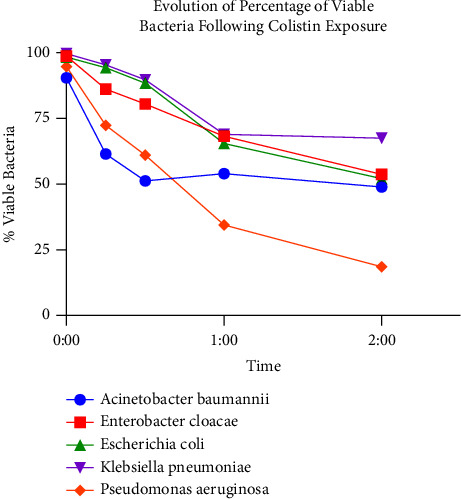
Evolution of percentage of viable bacteria following Colistin exposure. Observation of Colistin SEM-AST assay applied to Colistin-sensitive strains of five clinically relevant species over all experimental time points.

Figure 7 reveals the evolution of the mean percentage of viable bacteria in all Colistin-sensitive species tested, at successive time points. It is clear that a significant drop in the mean percentage of viable bacteria is observed as early as 30 minutes.

#### 3.2.7. Independent Operator Comparison

Upon comparison between gold standard detection and the ability of two independent SEM operators to detect the susceptibility profile of five randomly selected isolates, there was a 100% agreement for both methods. The observation was recorded at all time points and both operators reported the correct susceptibility profile depending on the morphological changes observed at the earliest time point, in agreement with our results.

## 4. Discussion

Our current work has enabled us to perform Colistin antimicrobial susceptibility testing on five clinically significant species by applying a novel rapid SEM-AST assay [10–12].

Using our novel assay, we were able to quantitatively determine the direct effect of exposing bacteria to the antibiotic Colistin, providing a simplified approach to determining bacterial susceptibility to this molecule limited to the description of whether an isolate is sensitive or resistant to Colistin. In fact, we identified a significant drop in the percentage of viable organisms starting 30 minutes postexposure in susceptible strains, as compared to nonsignificant changes in resistant strains across all tested organisms. Our observation matched 100% of the susceptibility profiles tested using the surrogate chosen for the gold standard BMD. This was further confirmed by blindly testing the susceptibility profile identification capacity of two independent operators who were able to identify whether or not the isolate processed using our SEM-AST assay was sensitive or resistant to Colistin.

The killing effect of Colistin was best observed after 120 minutes of incubation with the antibiotic, with significant changes in morphologic characteristics, including bacterial cell inflation, distortion, fusion, and lysis, observed as early as 30 minutes after antibiotic exposure. Therefore, our method paves the way for a direct phenotypic analysis of the interaction of Colistin with bacteria, allowing the identification of distinctive features between different species. Furthermore, observing morphological and contrast differences can provide us with a way to create a phenotypic fingerprint for bacteria exposed to Colistin, providing rapid information on both identification (to the genus level) and susceptibility profile. Some challenges remain pertinent, mainly related to the definition of a susceptible/intermediate profile for Colistin, which is debated between CLSI and EUCAST. Reporting guidelines on Colistin susceptibility differ widely. CLSI has proceeded to eliminating the susceptible profile and only provides break points for intermediate and resistant profiles [7]. Furthermore, our method allows detection of the susceptibility profile at a fixed MIC level, which is the concentration of antibiotics typically defined in the guidelines as the break point between sensitive and resistant strains. Further research is underway to expand the concentrations of tested antibiotics and integrate them into a single assay to provide a variable MIC result to further demonstrate the microbiological profile of the tested organism and provide information on therapeutic options.

Furthermore, due to its broad spectrum, Colistin has been widely used in the empiric treatment of septic patients who have a history of MDR infections, as well as in regions of significant prevalence of MDR [19]. This highlights the importance of rapid determination of susceptibility to Colistin in performing the appropriate selection of antibiotics early on. This is particularly challenging due to the fact that the broth microdilution method has multiple pitfalls related to the design of the test [5] and is not suitable for routine use in daily microbiology laboratory workflows [3]. Most traditional AST methods, including BMD, require long turnaround times (TAT) that reach 72 hours from sampling [20]. Over the past decade, there have been enormous advances in the development of AST techniques, and most importantly rapid AST methods. These developments have put in use a number of techniques such as turbidimetry, disk diffusion, modified microdilution, and molecular testing, among others [20]. However, all of these methods must provide results comparable to BMD and prove practical enough for use in routine clinical microbiology laboratory settings. These advances are truly multifaceted in that they take advantage of both phenotypic and genotypic characteristics of bacterial interaction with antibiotics. Interestingly, there have been several advances related to imaging-based methodologies that utilize optical sensors, bright-field microscopy, and combinations of optical sensors with other technologies [21]. Some of these techniques have allowed the determination of AST results in less than 5 hours, but remain difficult to disseminate as a replacement for BMD in routine practice due to performance issues of great importance and high cost [22, 23]. Overall, BMD remains the most reliable method for determining the susceptibility of Colistin. The recent implementation of automated antimicrobial susceptibility testing systems, such as VITEK 2 [24] and MicroScan [8], provided a promising approach to a faster AST, but these remain unreliable for Colistin. Molecular-based assays are also available, but are not a cost-effective option given the diversity of mechanisms associated with resistance to Colistin, including those that are not yet identified [3].

Our method allows for direct observation of the morphological changes in bacterial cells upon antibiotic exposure. Furthermore, the main advantage of our method is the ability to directly distinguish living bacteria from dead bacteria, assisted by PTA staining [11], within a well-defined concentration of organisms. This distinction of differences in bacterial cell contrast, coupled with a description of morphological changes, allows direct quantification of live and dead bacteria on the obtained micrographs, providing clear evidence of the effect of Colistin on the viability of the organisms, thus determining the susceptibility profile to Colistin. This work is the fourth application [10–12, 17] of our SEM-AST assay, confirming the promising results this assay provides compared to traditional AST assays. Future applications of this assay will include other antibiotics and continue to answer the simple question of whether a bacterial isolate is sensitive or resistant to a particular antibiotic. This will serve as the basis for future development of a database including specific morphologic features allowing for interspecies differentiation (for both identification and AST), which can then lead to the development of an artificial intelligence tool for a complete diagnostic solution using SEM.

In conclusion, we provide an extended proof of concept for the use of the SEM-AST assay with PTA for Colistin antimicrobial susceptibility testing for a number of clinically relevant bacterial species, providing a rapid and reliable susceptibility profile for a critical life-saving antibiotic.

## Figures and Tables

**Table 1:** Isolate listing from “*Collection de Souches de l'Unité des Rickettsies*” (*CSUR*) with MIC values and Colistin susceptibility.

Bacterial species	CSUR reference	MIC value (mg/L)	Colistin susceptibility
*Escherichia coli*	Q5584	0.5	Sensitive
*Escherichia coli*	Q8480	0.5	Sensitive
*Escherichia coli*	Q5583	0.25	Sensitive
*Escherichia coli*	Q5585	0.25	Sensitive
*Escherichia coli*	P6528	4	Resistant
*Escherichia coli*	P9544	8	Resistant
*Escherichia coli*	Q1065	8	Resistant
*Escherichia coli*	P4969	4	Resistant

*Klebsiella pneumoniae*	Q8478	0.5	Sensitive
*Klebsiella pneumoniae*	Q8476	0.25	Sensitive
*Klebsiella pneumoniae*	P9554	0.5	Sensitive
*Klebsiella pneumoniae*	Q8477	0.25	Sensitive
*Klebsiella pneumoniae*	P9552	0.125	Sensitive
*Klebsiella pneumoniae*	P1594	32	Resistant
*Klebsiella pneumoniae*	Q4757	4	Resistant
*Klebsiella pneumoniae*	P9555	4	Resistant
*Klebsiella pneumoniae*	P1591	8	Resistant
*Klebsiella pneumoniae*	P1593	32	Resistant

*Enterobacter cloacae*	Q5598	0.25	Sensitive
*Enterobacter cloacae*	Q5601	0.5	Sensitive
*Enterobacter cloacae*	Q5599	0.5	Sensitive
*Enterobacter cloacae*	Q5606	0.25	Sensitive
*Enterobacter cloacae*	Q5600	0.5	Sensitive
*Enterobacter cloacae*	Q0181	64	Resistant
*Enterobacter cloacae*	P6538	>64	Resistant
*Enterobacter cloacae*	Q3814	>64	Resistant
*Enterobacter cloacae*	Q8771	64	Resistant
*Enterobacter cloacae*	P9550	32	Resistant

*Acinetobacter baumannii*	Q8723	0.5	Sensitive
*Acinetobacter baumannii*	Q5578	1	Sensitive
*Acinetobacter baumannii*	Q5577	1	Sensitive
*Acinetobacter baumannii*	Q8724	0.25	Sensitive
*Acinetobacter baumannii*	Q7189	8	Resistant
*Acinetobacter baumannii*	Q7188	4	Resistant
*Acinetobacter baumannii*	Q8722	8	Resistant

*Pseudomonas aeruginosa*	P9560	0.25	Sensitive
*Pseudomonas aeruginosa*	Q8732	0.125	Sensitive
*Pseudomonas aeruginosa*	Q8721	0.5	Sensitive
*Pseudomonas aeruginosa*	Q8720	0.5	Sensitive
*Pseudomonas aeruginosa*	Q7185	0.25	Sensitive
*Pseudomonas aeruginosa*	Q7186	8	Resistant
*Pseudomonas aeruginosa*	Q8719	8	Resistant
*Pseudomonas aeruginosa*	Q8731	4	Resistant
*Pseudomonas aeruginosa*	Q8718	8	Resistant

## Data Availability

The data used to support the findings of this study are available from the corresponding author upon request.

## Supplementary Materials

Supplementary Materials Table S1: Colistin-EUCAST Epidemiological Cutoff for Selected Strains.
