# Supplementary material for: Antimicrobial Susceptibility Testing for Colistin: Extended Application of Novel Quantitative and Morphologic Assay Using Scanning Electron Microscopy
Source: Int J Microbiol. 2024 May 25;2024:8917136. doi: 10.1155/2024/8917136 (PMC11144066; doi:10.1155/2024/8917136)
Supplement: Supplementary Materials — Table S1: Colistin-EUCAST Epidemiological Cutoff for Selected Strains. [file 8917136.f1.docx]

**Supplementary Materials**

**Table S1: Colistin - EUCAST Epidemiological Cut-Off for Selected Strains**

|  | **Colistin - EUCAST Epidemiological Cut-Off** |
| --- | --- |
| *E. coli* | 2 mg/L |
| *K. pneumoniae* | 2 mg/L |
| *E. cloacae* | 2 mg/L |
| *A. baumannii* | 2 mg/L |
| *P. aeruginosa* | 4 mg/L |
